# Shotgun metagenomic analysis of the oral microbiome in gingivitis: a nested case-control study

**DOI:** 10.1080/20002297.2024.2330867

**Published:** 2024-03-22

**Authors:** Muhammed Manzoor, Jaakko Leskelä, Milla Pietiäinen, Nicolas Martinez-Majander, Eija Könönen, Teemu Niiranen, Leo Lahti, Juha Sinisalo, Jukka Putaala, Pirkko J. Pussinen, Susanna Paju

**Affiliations:** a Department of Oral and Maxillofacial Diseases, University of Helsinki, Helsinki, Finland; b Industrial Biotechnology and Food Protein Production, VTT Technical Research Centre of Finland, Espoo, Finland; c Department of Neurology, Helsinki University Hospital and University of Helsinki, Helsinki, Finland; d Institute of Dentistry, University of Turku, Turku, Finland; e Department of Public Health and Welfare, Finnish Institute for Health and Welfare, Helsinki, Finland; f Department of Internal Medicine, Turku University Hospital and University of Turku, Turku, Finland; g Department of Computing, University of Turku, Turku, Finland; h Heart and Lung Center, Helsinki University Central Hospital, and Helsinki University, Helsinki, Finland; i School of Medicine, Institute of Dentistry, University of Eastern Finland, Kuopio, Finland

**Keywords:** Metagenomics, oral microbiome, periodontal disease, chronic periodontitis, saliva, biomarker, dysbiosis, diversity, prediction

## Abstract

**Background:**

Gingivitis, i.e. inflammation of the gums, is often induced by dentalplaque. However, its exact link to the oral microbiota remains unclear.

**Methods:**

In a case-control study involving 120 participants, comprising 60 cases and 60 controls (mean age (SD) 36.6 (7.6) years; 50% males), nested within a prospective multicentre cohort study, we examined theoral microbiome composition of gingivitis patients and their controlsusing shotgun metagenomic sequencing of saliva samples. Participants underwent clinical and radiographic oral health examinations, including bleeding on probing (BOP), at six tooth sites. BOP ≥33%was considered ‘generalized gingivitis/initial periodontitis’(GG/IP), and BOP <33% as ‘healthy and localized gingivitis’(H/LG). Functional potential was inferred using HUMANn3.

**Results:**

GG/IP exhibited an increase in the abundance of *Actinomyces, Porphyromonas, Aggregatibacter, Corynebacterium, Olsenella, and Treponema*, whereas H/LG exhibited an increased abundance of *Candidatus Nanosynbacter*. Nineteen bacterial species and fourmicrobial functional profiles, including L-methionine, glycogen, andinosine-5’-phosphate biosynthesis, were associated with GG/IP. Constructing models with multiple markers resulted in a strong predictive value for GG/IP, with an area under the curve (ROC) of 0.907 (95% CI: 0.848-0.966).

**Conclusion:**

We observed distinct differences in the oral microbiome between the GG/IP and H/LG groups, indicating similar yet unique microbial profiles and emphasizing their potential role in progression of periodontal diseases.

## Introduction

Gingivitis is a pervasive oral health issue that affects a substantial portion of the global population. It is a prevalent periodontal disease in humans that typically arises from dental plaque, resulting in inflammation of gingival tissue [1]. Gingivitis is estimated to affect up to 90% of the world’s population at one time or another [2], and several epidemiological studies have estimated that the reported rates of gingivitis fluctuate significantly (ranging from 50% to 100%) among dentate patients in different populations [3,4]. The prevalence of gingivitis varies based on factors such as age, socio-demographic status, and oral hygiene habits. The main risk factors of gingivitis are poor oral hygiene, smoking, and tobacco use. Regular dental check-ups and maintenance of a good and healthy oral hygiene environment are important for both the prevention and management of gingivitis.

Gingival inflammation is clinically seen as bleeding on probing (BOP), as well as redness and swelling of the gingiva, without any signs of periodontal attachment loss. Gingivitis is a reversible condition that can be managed effectively with therapy and improved oral hygiene. However, if left untreated, it can progress to periodontitis, characterized by chronic inflammation and gum tissue deterioration, and ultimately, the loss of tooth attachment and alveolar bone [5,6]. The diagnosis of gingivitis long lacked a universal classification or clear-cut criteria to differentiate it from periodontal health. However, a classification system based on the BOP score has emerged [6]. Gingivitis is diagnosed when BOP is 10% or more, further classified into localized (BOP 10–30%) and generalized (BOP ˃30%) forms. Longitudinal clinical studies have shown that gingivitis is a risk factor for periodontitis [7,8]; however, the specific mechanisms by which the gingivitis-associated microbiota promote periodontitis remain unclear. Moreover, gingivitis results from a polymicrobial insult involving unidentified and uncultivated bacterial species as well as viruses, fungi, archaea, and parasites [9,10].

Oral microbiota plays a pivotal role in the development and progression of various oral and systemic diseases [11]. The oral microbiota, with its captivating complexity and diversity, introduces the concept of polymicrobial involvement in chronic oral infections. Most population-wide surveys of gingivitis-associated microbiota focusing on the ‘red complex’, which comprises *Porphyromonas gingivalis*, *Tannerella forsythia*, and *Treponema denticola* [12,13]. Although gingivitis precedes periodontitis [8], the extent to which health- and gingivitis-associated microbiota are protective and exclude pathobionts, as well as whether they precede and facilitate the growth of periodontitis-associated taxa, remains unclear [1,14].

Studies on the association between microbiota and gingivitis have shown inconsistent results in microbial communities owing to the different methods used and geographical variations [15]. Over the past decade, several studies have identified differences in the oral microbiome of gingivitis patients [16–20]. Stone et al. [18] observed changes in subgingival plaque microbiota during the resolution of naturally occurring gingivitis, with a shift towards healthier microbial species. Additionally, Wirth et al. [20] found differences in the relative abundance of *Akkermansia*, *Campylobacter, Fusobacterium*, and *Treponema* in supragingival plaque (biofilm) samples associated with gingivitis in adolescents. In a experimental gingivitis study, subgingival microbiota composition and periodontitis-associated species within the genera *Tannerella*, *Porphyromonas*, *Selenomonas*, and *Leptotrichia* were associated with gingivitis [19].

Despite numerous studies in the past decade highlighting the variations in the oral microbiome associated with gingivitis, the exact connection between the oral microbiome and gingivitis remains inconclusive. Most of these studies have utilized subgingival plaque samples and primarily relied on enumerating 16S amplicon sequences. Analysis of the saliva microbiota offers a straightforward collection method, reflects the oral microbiome, and has the potential to be a non-invasive diagnostic tool, making it a simple and beneficial way to identify different pathological processes [21]. Moreover, 16S sequencing methods have limitations in achieving comprehensive genomic coverage, increased data output, and improved bacterial and non-bacterial species detection capabilities [22]. Due to the limited availability of comprehensive metagenomic data pertaining to the oral cavity, we endeavoured to address this research gap through metagenomic analysis. Saliva samples obtained from individuals with generalized gingivitis/initial periodontitis (GG/IP) were examined meticulously. Subsequently, these samples were systematically juxtaposed with those collected from an age- and sex-matched control group exhibiting healthy/localized gingivitis (H/LG). This methodological approach was adopted to discern the distinctive microbial profiles and potential biomarkers associated with gingivitis and its progression to periodontitis.

## Materials and methods

### Study participants and design

Saliva samples were collected from participants in the SECRETO Oral study, a sub-study of the international, prospective, multicentre study entitled SECRETO (Searching for Explanations for Cryptogenic Stroke in the Young: Revealing the Etiology and Triggers) that enrolled young adults (age 18–49 years) presenting with an imaging-positive first-ever acute cryptogenic ischemic stroke. A comprehensive description of the study methods has been previously published [23].

A total of 329 subjects were enrolled in the SECRETO Oral study [24], and out of these, 120 participants were included in the present study. To be eligible for participation, individuals had to meet specific criteria: (1) undergo clinical oral examinations; (2) exhibit no radiographic bone loss; (3) refrain from antibiotic usage within one-month preceding saliva sampling; and (4) provide comprehensive information on gingivitis risk factors, such as smoking, alcohol consumption, and antibiotic use. We matched individuals from the GG/IP and H/LG groups, ensuring a balanced distribution of sex and age equivalence between the two groups. Body mass index (BMI) was determined by measuring their weight and height. Educational level was classified as primary/secondary or higher. Registered risk factors included a history of hypertension, diabetes mellitus, current or former tobacco smoking, alcohol consumption, and antibiotic use (within three months prior to the study visit).

### Clinical oral examination

Oral examinations for this study were conducted at the University of Helsinki and the University of Turku, Finland between April 2014 and February 2020. The examinations are described in detail elsewhere [24]. A standard dental office setting was used for clinical examinations that were conducted by the same periodontal specialist (SP). BOP and probing pocket depth (PPD) were assessed at six sites per tooth, and caries and number of missing teeth were registered. PPDs around partially erupted wisdom teeth were excluded. Panoramic tomography images were obtained from all participants to ensure that none of the participants had bone loss. Participants were classified into two groups: BOP score ≥ 33% was considered GG/IP, and BOP < 33% was considered H/LG [6].

### Metagenomic sequencing and bioinformatics analysis

Before the oral examination, participants chewed paraffin for approximately 5 min to stimulate a minimum of 4 ml of saliva, which was collected into RNase/DNase-free tubes and stored at − 70°C. Thawed saliva samples (500 µl) were mixed with 500 µl of lysis buffer (Chemagic DNA Blood 400-H96 Kit, PerkinElmer) in MN type B bead tubes (Macherey-Nagel). Subsequently, the samples were treated with 16 µl of proteinase K (20 mg/ml) and lysed with a Bead Ruptor Elite Homogenizer (Omni, Inc.) with three cycles of 1-min shaking (6 m/s) with a 5-min pause after each bead-beating period. The samples were incubated at 65°C for 1 hour and centrifuged at 8000 rpm for 1 min. 6 µl of RNaseA (10 mg/mL, Thermo Fisher Scientific) was added to 96 deep-well plates, and 600 µl of the supernatant was applied to the deep-well plates. DNA extraction was performed using a ChemagicTM 360 instrument with the Chemagic Saliva600 pre-filled protocol and a Chemagic DNA Blood 400-H96 Kit (PerkinElmer). DNA samples were eluted in 100 µl of elution buffer from the kit. DNA concentrations were measured using a DeNovix DS-11 Spectrophotometer/Fluorometer. For quality control of genomic DNA, we utilized the Agilent TapeStation 4200 system. DNA libraries were then prepared from 260 ng (10 ng/µl) of DNA using the NEBNext® Ultra™ II FS DNA Library preparation kit.

Shotgun metagenomic paired-end sequencing (2 × 150 bp) was conducted using an Illumina NovaSeq 6000. Quality control was done using FastQC (v. 0.11.9). Adapter sequences and adapters from the FASTQ were removed, and the remaining adapter remnants were trimmed using the Trimmomatic (v.0.39) [25]. KneadData (v. 0.10.0) was used to remove human-associated sequences through alignment with the human genome (GRCh38.p14). Duplicate reads were eliminated using BBMap (v. 38.91). Subsequently, taxonomy assignment was performed on the remaining reads using Kraken2 (v. 2.1.2) and species-level abundance estimates were refined using Bracken (v. 2.7) [26]. We constructed functional profiles for each sample based on the MetaCyc pathway database (https://metacyc.org/) using HUMAnN3 (v. 3.6) with default settings and the standard HuMAnN databases [27].

## Statistical analysis

All statistical analyses of microbiome data were conducted using R software (v. 4.2.2). Differences in characteristics and oral parameters between cases and controls were examined using the Wilcoxon signed-rank, Pearson χ2 test and McNemar tests.

For microbiome analysis, we employed microbiome-specific data containers in R, such as phyloseq [28] and TreeSummarizedExperiment [29]. Initially, we applied quality control filtering to the taxonomic data, utilizing a prevalence cut-off of 25% and a relative abundance threshold of 0.001%, resulting in the retention of 1846 microbial species. We removed taxonomic features with < 10 sequence reads before analysis. Alpha diversity indices, such as observed species and the Faith’s phylogenetic diversity (Faith’s PD: a phylogenetic generalization of species richness), were calculated using the R-package ‘vegan’ (v. 2.6.4) [30]. Differences in beta diversity between cases and controls were assessed using the Permutational Analysis of Variance (PERMANOVA; function ‘adonis2’) from the R-package ‘vegan’ (v. 2.6.4). To depict the diversity in taxonomic composition, we employed principal coordinate analysis (PCoA) based on the Bray-Curtis dissimilarity matrix and Jaccard dissimilarity.

We identified differentially abundant taxonomic features between cases and controls using the Linear Discriminant Analysis (LDA) Effect Size (LEfSe) [31]. Permutation tests were conducted across various taxonomic levels (phylum, genus, and species), and significant taxonomic features were selected using the Kruskal-Wallis test and LDA. Taxonomic features with *p*-values below 0.05 and LDA scores exceeding 2 were considered statistically significant. Functionally enriched features in the metagenomes were identified using the R package MaAsLin2 (v. 1.12.0) [32]. A statistically significant result was defined as an FDR-adjusted *p*-value <0.05.

Utilizing the differentially abundant microbial features identified by LEfSe, we used C-statistics to build predictive models for GG/IP and used area under the receiver operating characteristic (ROC) to present the results [33]. The AUC was measured using SPSS software (v.29.0.1.0) (SPSS Inc., Armonk, NY, USA). The models were trained using five-fold cross-validation on a dataset comprising 80% of the randomly selected samples. The model that exhibited the best performance was selected on the basis of the maximum AUC. The optimal model was subsequently evaluated and compared using validation datasets that comprised the remaining 20% of the samples. The crude model (Model 1) comprised all significant microbial features, including the identified species and pathways. Model 2 was additionally adjusted for caries and number of periodontal pockets. Model 3 was created by refining it through backward elimination.

## Results

### Characteristics of study participants

After applying exclusion criteria and accounting for occasional missing data, 60 patients with GG/IP and 60 age- and sex-matched H/LG were included in our analyses. Both groups had an even distribution of sex, with 50% males and 50% females (Table 1). The mean age (SD) was similar between cases and controls (GG/IP, 36.66 (7.61) vs. H/LG, 36.61 (7.62); *p* = 0.885). More than half of the participants (61.7%) had a high educational status, and 35.8% were smokers, but the frequencies did not differ between cases and controls. There was a higher prevalence of caries among cases (46.7%) than among controls (30.0%), although this difference was not significant (*p* = 0.078). GG/IP patients had a higher number of periodontal sites with PPD ≥4 mm and teeth affected by periodontal pockets (≥4 mm, *p* < 0.001) (Table 1).

**Table 1.** Characteristics of study subjects.

			GG/IP (*n* = 60)	H/LG (*n* = 60)	*p*-value
Gender, males (%)			30 (50)	30 (50)	1.000
Mean age (SD), years			36.66 (7.61)	36.61 (7.62)	0.885
BMI (SD), kg/m^2^			27.35 (6.13)	26.08 (5.08)	0.482
Higher education^#^ (%)			35 (58.3)	39 (65.0)	0.571
Smoking		Ever (%)	21 (35.0)	22 (36.7)	1.000
		Current (%)	6 (10.0)	4 (6.7)	0.759
Diagnosed diabetes (%)			1 (1.7)	4 (6.7)	0.371
Hypertension (%)			12 (20)	10 (16.7)	0.803
Heavy alcohol consumption (%)			11 (18.3)	9 (15.0)	0.789
Antibiotics use^$^, yes (%)			1 (1.7)	5 (8.3)	0.134
Statin medication^†^ (%)			5 (8.3)	1 (1.7)	0.221
Regular dentist check-ups (%)			32 (53.3)	41 (68.3)	0.164
Caries, yes (%)			28 (46.7)	18 (30.0)	0.078
Mucosal lesions and changes, yes (%)			4 (6.7)	5 (8.3)	1.000
No. of missing teeth (%)		0	44 (73.3)	46 (76.7)	0.702
		1–2	12 (20.0)	6 (10.0)	
		>2	4 (6.7)	8 (13.3)	
No. of teeth with periodontal pockets (≥4 mm) (%)		0–2	19 (31.7)	41 (68.3)	**<0.001**
		3–9	32 (53.3)	19 (31.7)	
		≥10	9 (15.0)	0 (0)	
No. of periodontal pockets (≥4 mm) (%)		0–5	30 (50.0)	52 (86.7)	**<0.001**
		6–19	25 (41.7)	8 (13.3)	
		≥20	5 (8.3)	0 (0)	

^#^Post-secondary non-tertiary education or tertiary education, ^$^Prescribed antibiotic treatment within three months prior to oral examination. ^†^Current medication registered from the recruitment time. GG/IP: generalized gingivitis/initial periodontitis, H/LG: healthy/localized gingivitis.

### Alpha and beta diversity

**Figure 1.**
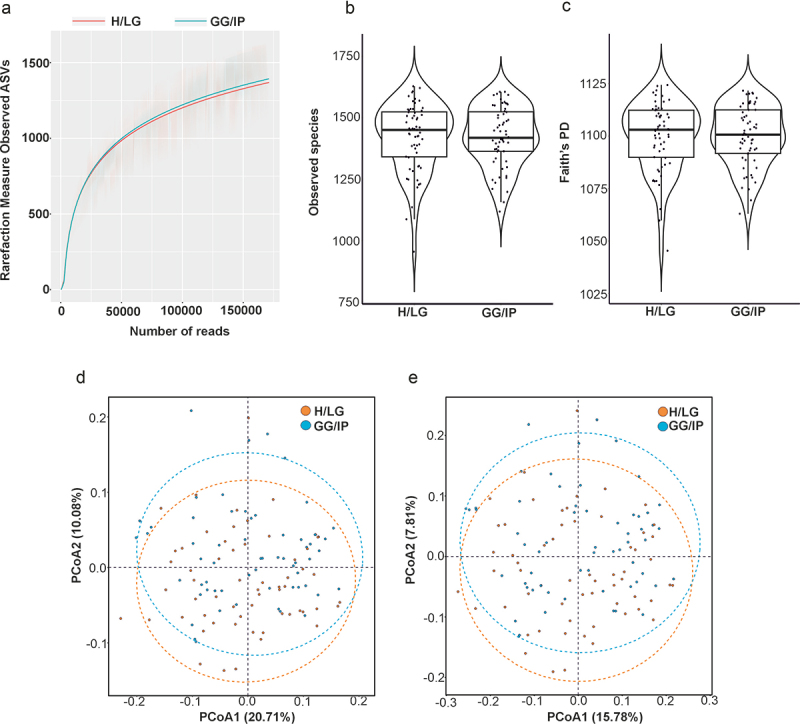
Alpha and beta diversity measurements of the oral microbiome. (a) Rarefaction curve constructed based on observed amplicon sequence variants (ASVs). Alpha diversity measurements in terms of (b) observed species and (c) Faith’s phylogenetic diversity indices. Principal coordinate analysis (PCoA) of beta diversity plots based on (d) Bray – Curtis and (e) Jaccard indices. H/LG: healthy/localized gingivitis, GG/IP: generalized gingivitis/initial periodontitis.

A total of 20 420 927 high-quality reads and 1846 unique amplicon sequence variants (ASVs) were identified at the species level in the 120 samples. The rarefaction curve constructed based on the observed ASVs indicated that the sample quality was high and the sample size was reasonable (Figure 1(a)). Alpha diversity indices did not differ between cases and controls regarding observed species (*p* = 0.790, Figure 1(b)) or Faith’s PD (*p* = 0.830, Figure 1(c)).Although a slight variance in the microbial community composition (beta diversity) was observed between cases and controls, this disparity was not significant (PERMANOVA, Bray-Curtis dissimilarity: F = 1.771, *p* = 0.076, Figure 1(d); Jaccard dissimilarity: F = 1.393, *p* = 0.121, Figure 1(e); Table S1).

### Composition of the oral microbiota

**Figure 2.**
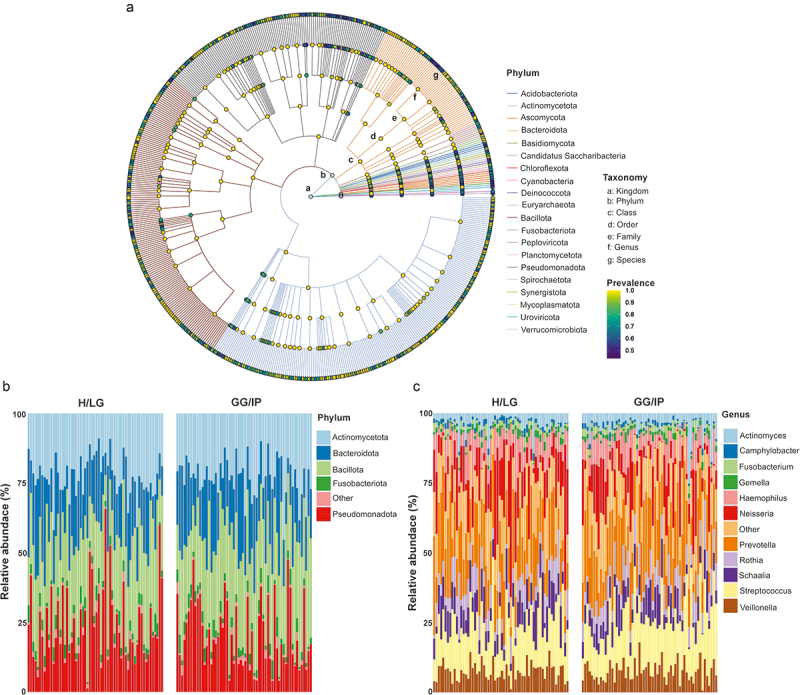
Differences in oral microbial communities of GG/IP and H/LG. (a) Metagenomics-based phylogenetic tree showing the prevalence of top phyla based on mean abundance. Moving from the inner to outer circles, the taxonomic hierarchy spans from kingdom to species. Node diameter reflects abundance across various taxonomic levels, while distinct colours represent different taxonomic clades. Stacked bar plot of the mean relative abundance of bacterial taxa at the (b) phylum and (c) genus levels in the GG/IP and H/LG groups, revealing distinctive community compositions. H/LG: healthy/localized gingivitis, GG/IP: generalized gingivitis/initial periodontitis.

We constructed a metagenomics-based phylogenetic tree for the most abundant phyla, allowing observation of their taxonomic composition and abundance (Figure 2(a)). The oral microbiota comprised 1846 microbial species distributed among 21 phyla. To visualize the differences in microbial composition between cases and controls, bar plots displaying the relative bacterial abundance were created at both the phylum and genus levels. As shown in Figure 2(b), more than 95% of the representative sequences were assigned to five major phyla: Bacillota (Firmicutes), Actinomycetota (Actinobacteria), Pseudomonadota (Proteobacteria), Bacteroidota (Bacteroidetes), and Fusobacteriota (Fusobacteria). Bacillota and Bacteroidota predominated in cases, whereas Pseudomonadota and Actinomycetota were higher in controls (Figure. S1A, Table S2). A marked increase in the abundance of the phylum Spirochaetota and a decrease in the abundance of *Candidatus* Saccharibacteria (formerly known as TM7), were observed in cases (*p* < 0.05) (Table S3).

**Figure 3.**
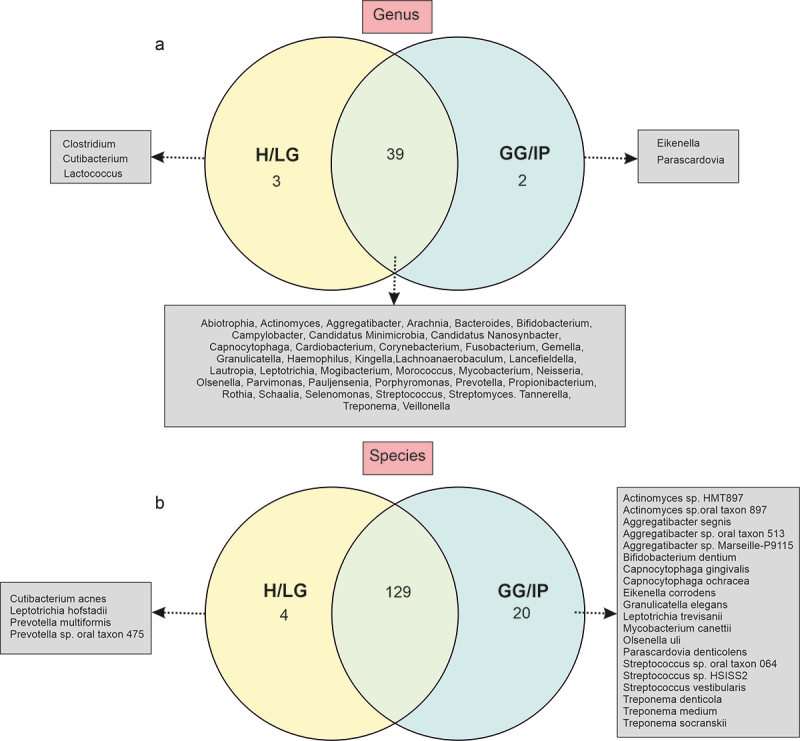
Venn diagram depicting shared oral microbiota among the GG/IP and H/LG groups, including only taxa at (a) genus and (b) species levels, present in at least 90% of samples in each group. H/LG: healthy/localized gingivitis, GG/IP: generalized gingivitis/initial periodontitis.

**Figure 4.**
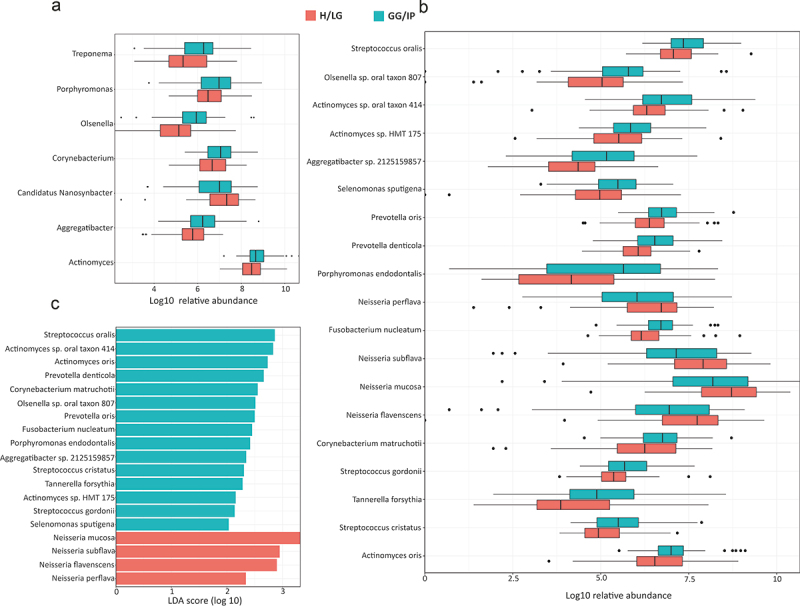
Differences in the oral microbiota composition between GG/IP and H/LG using the LEfSe analysis. (a) Boxplot showing the taxonomic abundance of significant bacterial genera in GG/IP and H/LG. (b) Boxplot showing the taxonomic abundance of significant bacterial species in GG/IP and H/LG. (c) Histogram showing LDA distribution (LDA score > 2) of species abundance of oral microbiota in the two groups. H/LG: healthy/localized gingivitis, GG/IP: generalized gingivitis/initial periodontitis.

*Prevotella, Streptococcus*, and *Veillonella* were the predominant genera in cases, whereas the relative abundances of *Neisseria, Schaalia*, and *Haemophilus* were higher in controls (Figure 2(c) and S1B). Core genera and species shared between cases and controls in at-least 90% of samples are presented in Figure 3; the Venn diagram show that of the 44 identified core genera, 39 were common to both cases and controls (Figure 3(a)). Similarly, among the 153 core species, 129 species were found in both cases and controls (Figure 3(b)). At the genus level, an increase in *Actinomyces, Porphyromonas, Aggregatibacter, Corynebacterium, Olsenella*, and *Treponema* abundances was observed in cases, and an increased abundance of *Candidatus* Nanosynbacter occured in controls (*p* < 0.05) (Figure 4(a), Table S3).

### Comparison of microbial biomarkers

Consistent with the genus-level results, we found that 19 bacterial species were associated with GG/IP (Figure 4(b)). LEfSe analysis showed enrichment of *Actinomyces* (sp. oral taxon 414 sp. HMT 175, and *A. oris*), *Streptococcus* (*S. oralis, S. cristatus* and *S. gordonii*), *Prevotella* (*P. denticola* and *P. oris*), *Corynebacterium matruchotii, Olsenella* sp. oral taxon 807, *Fusobacterium nucleatum, Porphyromonas endodontalis, T. forsythia, Aggregatibacter* sp. 2125159857, and *Selenomonas sputigena* in cases compared with controls (*p* < 0.05). The relative abundance of several *Neisseria* species, including *N. mucosa, N. subflava, N. flavescens*, and *N. perflava*, were lower in cases than in controls (*p* < 0.05) (Figure 4(c)).

### Functional profiling

Considering contributions of individual species, we discovered four metagenomic pathways that exhibited significant differences in abundance between cases and controls (original *p* < 0.05, *q* < 0.25) (Table S4). Among these pathways, GG/IP patients showed increased abundance in pathways associated with L-methionine biosynthesis III (HSERMETANA-PWY), inosine-5'-phosphate (5'-IMP) biosynthesis (PWY-6124), tRNA charging (TRNA-CHARGING-PWY), and glycogen biosynthesis I (from ADP-D-Glucose) (GLYCOGENSYNTH-PWY).

### Oral-microbiome-based predictive models

**Figure 5.**
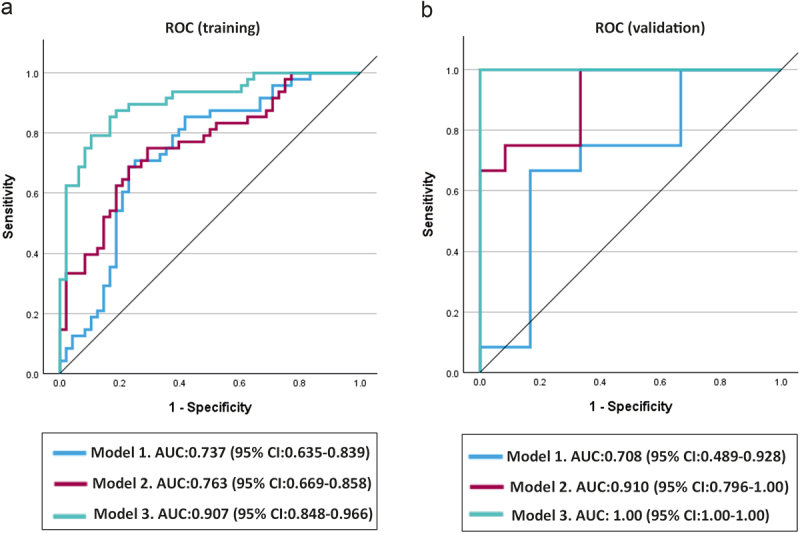
Receiver operating characteristic (ROC) curve of microbial species biomarkers associated with GG/IP based on microbial abundance. (a) The curve illustrates the ROC curve of microbial species biomarkers associated with GG/IP in the training model. The models were trained on a dataset comprising 80% of the randomly selected samples, including 96 individuals with an equal distribution of 48 participants (50%) in both the H/LG and GG/IP groups. (b) The ROC curve illustrates the validation models, which comprised the remaining 20% of the samples. Model 1 included all significant microbial features. Model 2 was additionally adjusted for caries and number of periodontal pockets. Model 3 comprised seven features, including five bacterial species, along with the covariates of caries and number of periodontal pockets.

The predictive capacity of significant features for GG/IP was assessed in both the training and validation datasets (Table S5). The initial model (Model 1) was built using all significant bacterial species, resulting in an AUC of 0.737 (95% CI: 0.635–0.839) for the training dataset and 0.708 (95% CI: 0.489–0.928) for the validation dataset. Incorporating caries and the number of periodontal pockets into Model 1 increased the predictive capability to an AUC of 0.763 (95% CI: 0.669–0.858) for the training dataset and 0.910 (95% CI: 0.796–1.00) for the validation dataset. Refining the model further strengthened its predictive value for GG/IP, achieving an AUC of 0.907 (95% CI: 0.848–0.966) for the training dataset and 1.00 (95% CI: 1.00–1.00) for the validation dataset (Figure 5). The final prediction model consisted of seven features, including five bacterial species *(P. denticola, S. oralis, Aggregatibacter* sp. 2125159857, *S. cristatus* and *S. sputigena*) along with the covariates of caries and the number of periodontal pockets.

## Discussion

Using a thoroughly characterized cohort of young and early middle-aged individuals and their age- and sex-matched pairs nested within a prospective multicentre cohort study, we observed that the oral microbiota is associated with the risk of gingivitis. We found that the oral microbiomes in GG/IP patients were dominated by the genera *Actinomyces*, *Aggregatibacter*, *Porphyromonas*, *Corynebacterium*, *Olsenella*, and *Treponema*. Constructing models with multiple markers resulted in a strong predictive value for GG/IP. We noted an association between 19 bacterial species and GG/IP, supporting their pathogenic role in the initiation/progression of periodontitis.

Microbial diversity in terms of alpha and beta diversity in the saliva of individuals with GG/IP and H/LG remained unchanged. Similarly, Deng et al. [34] reported no notable differences in alpha diversity between patients with gingivitis and controls. This is consistent with another study that used both 16S rRNA gene sequencing and whole metagenome sequencing to compare saliva and dental plaque samples of individuals diagnosed with spontaneous gingivitis caused by plaque, induced gingivitis in patients wearing fixed metal braces, and healthy individuals without symptoms of gingivitis [20].

The overall diversity and composition of the oral microbiota showed similarities between cases and controls. While variations in the frequency of detecting taxa existed between cases and controls, the majority of GG/IP-associated taxa were also found in H/LG but in lower proportions. In our study, the most commonly found species belonged to the major phyla in the human oral microbiota, including Bacillota, Actinomycetota, Pseudomonadota, Bacteroidota, and Fusobacteriota [35]. None of these phyla differed between cases and controls. However, the phylum Spirochaetota was relatively more abundant and *Candidatus* Saccharibacteria less abundant in cases. Previous studies have demonstrated a higher relative abundance of Spirochaetota in patients with ulcerative gingivitis and chronic periodontitis than in healthy controls [36,37]. Spirochaetota, including species of the genus *Treponema*, can benefit from interactions with other community members, potentially resulting in their dominance through autogenic succession [38]. However, further studies are needed to understand the specific reasons for the dominance of Spirochaetota in gingivitis [37]. *Candidatus* Saccharibacteria, a member of the human oral microbiome belonging to the Candidate Phyla Radiation (CPR), primarily acts as a commensal and can modulate the microbial environment by inhibiting the growth of other bacteria [39]. Kharitonova et al. [40], demonstrated that the shift from healthy controls to localized initial periodontitis in young adults might be linked to a reduction in the diversity and abundance of some members of the phyla *Candidatus* Saccharibacteria, supporting our findings.

The most abundant genera found in the oral cavity in this study were *Prevotella, Streptococcus*, and *Neisseria*, consistent with earlier findings [41]. *Actinomyces, Aggregatibacter, Porphyromonas, Corynebacterium, Olsenella*, and *Treponema* were significantly higher, whereas *Candidatus* Nanosynbacter was less abundant in cases than in controls. Our findings align with the previously suggested roles of *Actinomyces, Aggregatibacter, Porphyromonas*, and *Treponema* in gingivitis pathogenesis [42–44]. However, the relatively less prevalent *Candidatus Nanosynbacter* requires further examination [45]. In contrast, some genera, including *Neisseria*, and *Haemophilus* were more abundant in H/LG and these genera are associated with gingival health [19,46].

Analysis of the oral microbiota composition revealed that 15 species were more abundant, while four species were less abundant in cases than in controls. Among the significantly abundant species, *P. denticola* exhibited the best performance in discriminating between cases and controls (Table S5). This finding significantly reinforces prior research highlighting the role of *P. denticola* in inflammation and the breakdown of gum tissue [47], emphasizing its potential significance as a contributing factor in the transition from gingivitis to more severe periodontal conditions [48]. *S. oralis, F. nucleatum*, *T. forsythia*, and *A. oris* were significantly higher in cases than in controls. The significant abundance of *S. oralis* in our study supports the hypothesis that this species is the predominant colonizer in the early stages of biofilm formation [49,50]. *F. nucleatum* can co-aggregate with both early and late colonizers in the oral cavity to form complexes that associate with each other in a virulent manner, thereby contributing to periodontal disease progression [51,52]. We previously demonstrated an association between increased levels of serum IgA/IgG antibodies specific to *F. nucleatum* in patients with active periodontitis with high BOP displaying elevated subgingival microbial levels [53]. In the present study, *T. forsythia*, a red complex bacterial species, showed a significant difference between cases and controls. A recent study using saliva 16S rRNA gene sequencing suggested that *T. forsythia* could be considered a candidate biomarker for distinguishing the severity of periodontal disease [54]. We previously reported a significant association between high *T. forsythia* saliva concentration and the number of periodontal pockets and BOP [55]. *Actinomyces* species have been established as early colonizers of dental plaque maturation, significantly contributing to gingivitis [56]. Additionally, *A. oris* was more frequently identified in the subgingival plaque of individuals with periodontitis than in healthy controls [57], supporting our findings. The results of the present study were also consistent with a recent study that reported an increase in the abundance of the red complex in the subgingival microbiome of patients with gingivitis compared to periodontally healthy subjects [58]. According to previous studies, most periodontitis-related species are more abundant in the gingivitis group than in the periodontally healthy group, supporting our findings [59].

Another significant finding of this study is the higher prevalence of many *Neisseria* species, including *N. mucosa, N. subflava, N. flavescens*, and *N. perflava*, in the H/LG group. *Neisseria* is a highly abundant genus that colonizes the oral cavity and has been the subject of studies assessing its protective role against many oral diseases [60]. Studies showing the predominance of *Neisseria* in the oral microbiome indicate healthy periodontal conditions [61,62], which is consistent with our observations.

Results from the pathway analyses further revealed that the oral microbiome and GG/IP risk, possibly through its involvement in the biosynthesis of L-methionine, 5'-IMP, and glycogen. 5'-IMP plays a central role in intracellular nucleotide synthesis and also regulates inflammation [63]. Methionine is obtained through the diet and is synthesized by several biochemical reactions in the body. Disruptions in methionine metabolism have been observed in various diseases such as cancer [64]. However, the connection between the oral microbiota and its involvement in the production of L-methionine, as well as its relationship to GG/IP, remains unclear.

Strengths of our study include a carefully planned design, an assessment subset that was age- and sex-matched, and a detailed analysis of periodontal conditions. Additionally, utilizing shotgun metagenomic sequencing expanded genomic coverage, yielding more data, enabling the detection of various microbial species, and predicting pathways [22]. Limitations of the study include the single-time sample collection and the relatively small sample size, which affected the depth of our analysis and hindered our ability to assess the impact of oral microbiota changes over time. The diagnosis of GG/IP can be deemed a limitation due to the insufficient number of subjects for distinct categorization into H/LG and GG/IP groups. Consequently, localized and incipient gingivitis were incorporated into the H/LG group. Additionally, the threshold that we employed to categorize GG/IP groups was a BOP value of 33%, which slightly exceeded the consensus (BOP >30%) [6]. Our findings indicate that young and early middle-aged Finnish adults frequently omit the use of interdental cleaning devices (brush and floss) from their daily oral hygiene routines.

In our study, we conducted microbiome assessments using saliva samples. Nonetheless, a sampling strategy focusing on analysing the microbiome from other oral niches, such as selected sites with sub- or supragingival plaque, might lead to an incomplete description of participants’ oral microbiome profile. Moreover, the utilization of mean values to represent the relative microbial abundance in our data is a limitation. However, many studies have noted the non-normal distribution observed in microbiota data, and the presence of outliers can significantly impact microbial abundance [65]. Moreover, our study enrolled only young and early middle-aged individuals. However, previous studies have reported age-related changes in the oral microbiome composition [66,67]. Although we successfully excluded participants using antibiotics less than one month prior to saliva sampling, 5% of them had used antibiotics in the past 3 months, introducing a potential influence on the composition of the oral microbiome. Long-term antibiotic use can have prolonged effects on the salivary microbiome, thereby influencing oral health and potentially contributing to dysbiosis [68]. Therefore, further large prospective studies are necessary to understand the significance of these unique microbial biomarkers in the initiation and/or progression of periodontitis.

## Conclusions

Our study highlights a distinct difference in the oral microbiome composition between individuals with GG/IP and H/LG. We demonstrated that constructing models with multiple markers resulted in a strong predictive capability for gingivitis. The increased relative abundance of specific bacterial taxa in the saliva of individuals with GG/IP suggests their role in disease progression and potential diagnostic value in preventing further advancement toward periodontitis.

## Supplementary Material

Supplemental Files.docx

## Data Availability

The data supporting the findings of this study are available upon request from the corresponding author.
