# Supplementary material for: Shotgun metagenomic analysis of the oral microbiome in gingivitis: a nested case-control study
Source: J Oral Microbiol. 2024 Mar 22;16(1):2330867. doi: 10.1080/20002297.2024.2330867 (PMC10962305; doi:10.1080/20002297.2024.2330867)
Supplement: Supplemental Files.docx [file ZJOM_A_2330867_SM2473.docx]

**Shotgun metagenomic analysis of the oral microbiome in gingivitis: A nested case-control study**

**Supplemental Tables**

**Table S1.** Dissimilarity tests of microbial community composition between GG/IP and H/LG were performed using Permutational Multivariate Analysis of Variance (PERMANOVA) based on Bray-Curtis and Jaccard distances. PERMANOVA: permutational multivariate analysis of variance.

| **PERMANOVA** | | | | |
| --- | --- | --- | --- | --- |
| **Bray-Curtis dissimilarity** | SumOfSqs | R^2^ | F-value | *p*-value |
|  | 0.164774 | 0.0148 | 1.771 | 0.076 |
| **Jaccard dissimilarity** | | | | |
|  | 0.243433 | 0.0117 | 1.393 | 0.121 |

**Table S2.** Relative abundance (mean (SD), %) of the most common phyla and genera in both cases and controls.

|  | H/LG | GG/IP |
| --- | --- | --- |
| **Phylum** |  |  |
| Bacillota | 28.85 (8.28) | 31.43 (8.54) |
| Pseudomonadota | 23.96 (13.75) | 20.28 (11.89) |
| Actinomycetota | 22.73 (6.92) | 22.18 (6.86) |
| Bacteroidota | 20.33 (8.18) | 21.05 (8.51) |
| Fusobacteriota | 3.1 (1.56) | 3.05 (1.68) |
| **Genus** |  |  |
| *Prevotella* | 18.07 (8.36) | 18.68 (8.89) |
| *Streptococcus* | 16.36 (6.85) | 18.88 (7.22) |
| *Neisseria* | 13.03 (11.47) | 9.83 (9.08) |
| *Schaalia* | 8.48 (4.32) | 7.28 (3.92) |
| *Veillonella* | 7.51 (3.15) | 7.57 (3.59) |
| *Haemophilus* | 7.63 (4.51) | 7.09 (3.84) |
| *Rothia* | 6.83 (3.81) | 7.06 (3.38) |
| *Actinomyces* | 3.63 (2.89) | 4.64 (4.08) |
| *Fusobacterium* | 1.76 (1.05) | 1.76 (1.08) |
| *Gemella* | 1.41 (0.86) | 1.40 (0.83) |

H/LG: healthy and localized gingivitis, GG/IP: generalized gingivitis/initial periodontitis.

**Table S3.** LEfSe results listing the most differentially abundant phyla, genera and species between GG/IP and H/LG groups.

|  | **Feature** | **Enriched group** | **Ef LDA** | **p-adj** |
| --- | --- | --- | --- | --- |
| **Phylum** | *Candidatus* Saccharibacteria | H/LG | 2.758 | 0.030 |
|  | Spirochaetota | GG/IP | 2.511 | 0.001 |
| **Genus** | *Candidatus* Nanosynbacter | H/LG | 2.642 | 0.039 |
|  | *Actinomyces* | GG/IP | 3.248 | 0.041 |
|  | *Porphyromonas* | GG/IP | 2.722 | 0.029 |
|  | *Aggregatibacter* | GG/IP | 2.686 | 0.001 |
|  | *Corynebacterium* | GG/IP | 2.636 | 0.041 |
|  | *Olsenella* | GG/IP | 2.535 | 1.56E-05 |
|  | *Treponema* | GG/IP | 2.497 | 0.002 |
| **Species** | *Neisseria mucosa* | H/LG | 3.322 | 0.046 |
|  | *Neisseria subflava* | H/LG | 2.949 | 0.035 |
|  | *Neisseria flavescens* | H/LG | 2.899 | 0.026 |
|  | *Neisseria perflava* | H/LG | 2.342 | 0.049 |
|  | *Streptococcus oralis* | GG/IP | 2.863 | 0.007 |
|  | *Actinomyces* sp. oral taxon 414 | GG/IP | 2.833 | 0.008 |
|  | *Actinomyces oris* | GG/IP | 2.735 | 0.012 |
|  | *Prevotella denticola* | GG/IP | 2.664 | 0.000 |
|  | *Corynebacterium matruchotii* | GG/IP | 2.556 | 0.021 |
|  | *Olsenella* sp. oral taxon 807 | GG/IP | 2.513 | 0.000 |
|  | *Prevotella oris* | GG/IP | 2.501 | 0.005 |
|  | *Fusobacterium nucleatum* | GG/IP | 2.454 | 0.000 |
|  | *Porphyromonas endodontalis* | GG/IP | 2.419 | 0.002 |
|  | *Aggregatibacter* sp. 2125159857 | GG/IP | 2.349 | 0.001 |
|  | *Streptococcus cristatus* | GG/IP | 2.307 | 0.001 |
|  | *Actinomyces* sp. HMT 175 | GG/IP | 2.159 | 0.043 |
|  | *Streptococcus gordonii* | GG/IP | 2.140 | 0.008 |
|  | *Selenomonas sputigena* | GG/IP | 2.033 | 0.005 |
| **Red complex bacteria** | *Porphyromonas gingivalis* | GG/IP | 2.090 | 0.040 |
|  | *Treponema denticola* | GG/IP | 1.840 | 0.005 |
|  | *Tannerella forsythia* | GG/IP | 2.283 | 0.001 |

Ef LDA = Linear Discriminant Analysis score or effect size for discriminative biomarker. *p*–adj: Benjamini-Hochberg adjusted *p*-value. H/LG: healthy and localized gingivitis, GG/IP: generalized gingivitis/initial periodontitis.

**Table S4**. List of MetaCyc pathways significantly altered between GG/IP and H/LG (original *q* < 0.25, Wilcoxon rank-sum test).

| **MetaCyc pathway** | **MetaCyc ID** | **Species attribution** | **Enriched group** | **coef** | ***p*–adj** |
| --- | --- | --- | --- | --- | --- |
| L-methionine biosynthesis III | HSERMETANA-PWY | *Actinomyces* sp. ICM47 | GG/IP | 0.0024 | 0.218 |
| Inosine-5'-phosphate biosynthesis | PWY-6124 | *Rothia dentocariosa* | GG/IP | 0.0021 | 0.219 |
| tRNA charging | TRNA-CHARGING-PWY | Unclassified | GG/IP | 0.0041 | 0.218 |
| Glycogen biosynthesis I (from ADP-D-Glucose) | GLYCOGENSYNTH-PWY | *S. cristatus* | GG/IP | 0.0021 | 0.239 |

**Table S5**. Classification of gingivitis status based on functional composition of the oral microbiome. The table displays AUC values for identified significant bacterial species.

|  | Model 1 (Training) | Model 2 (Training) | Model 1 (validation) | Model 2 (validation) |
| --- | --- | --- | --- | --- |
| *S. oralis* | 0.647 (0.537-0.757) | 0.752 (0.656-0.849) | 0.642 (0.410-0.874) | 0.896 (0.771-1.00) |
| *Actinomyces* sp. oral taxon 414 | 0.640 (0.529-0.751) | 0.756 (0.661-0.851) | 0.639 (0.406-0.872) | 0.924 (0.819-1.00) |
| *A. oris* | 0.640 (0.526-0.753) | 0.753 (0.656-0.850) | 0.618 (0.384-0.852) | 0.889 (0.761-1.00) |
| *P. denticola* | 0.743 (0.645-0.842) | 0.804 (0.715-0.893) | 0.486 (0.244-0.728) | 1.00 (1.00-1.00) |
| *C. matruchotii* | 0.618 (0.505-0.731) | 0.756 (0.660-0.852) | 0.618 (0.386-0.850) | 0. 910 (0.797-1.00) |
| *Olsenella* sp. oral taxon 807 | 0.688 (0.582-0.794) | 0.784 (0.695-0.874) | 0.705(0.484-0.926) | 0. 896 (0.771-1.00) |
| *P. oris* | 0.663 (0.554-0.771) | 0.752 (0.656-0.848) | 0.583 (0.345-0.822) | 0. 931 (0.833-1.00) |
| *F. nucleatum* | 0.714 (0.609-0.819) | 0.758 (0.653-0.852) | 0.674 (0.438-0.909) | 0. 931 (0.833-1.00) |
| *P. endodontalis* | 0.652 (0.541-0.762) | 0.749 (0.652-0.845) | 0.663 (0.421-0.905) | 0. 896 (0.771-1.00) |
| *Aggregatibacter* sp. 2125159857 | 0.700 (0.595-0.804) | 0.792 (0.703-0.881) | 0.559(0.313-0.805) | 0. 910 (0.796-1.00) |
| *S. cristatus* | 0.700 (0.597-0.804) | 0.798 (0.710-0.887) | 0.625 (0.389-0.861) | 0. 896 (0.773-1.00) |
| *T. forsythia* | 0.680 (0.572-0.788) | 0.746 (0.648-0.843) | 0.698 (0.477-0.919) | 0.910 (0.796-1.00) |
| *Actinomyces* sp. HMT 175 | 0.595 (0.480-0.711) | 0.739 (0.641-0.837) | 0.653 (0.428-0.877) | 0.896 (0.771-1.00) |
| *S. gordonii* | 0.664 (0.556-0.772) | 0.749 (0.651-0.847) | 0.573 (0.339-0.807) | 0.903 (0.778-1.00) |
| *S. sputigena* | 0.658 (0.550-0.766) | 0.798 (0.712-0.885) | 0.597 (0.349-0.846) | 0.924 (0.820-1.00) |
| All bacterial species | 0.737 (0.635-0.839) | 0.763 (0.669-0.853) | 0.708 (0.489-0.928) | 0.910 (0.796-1.00) |

**Supplemental Figures**


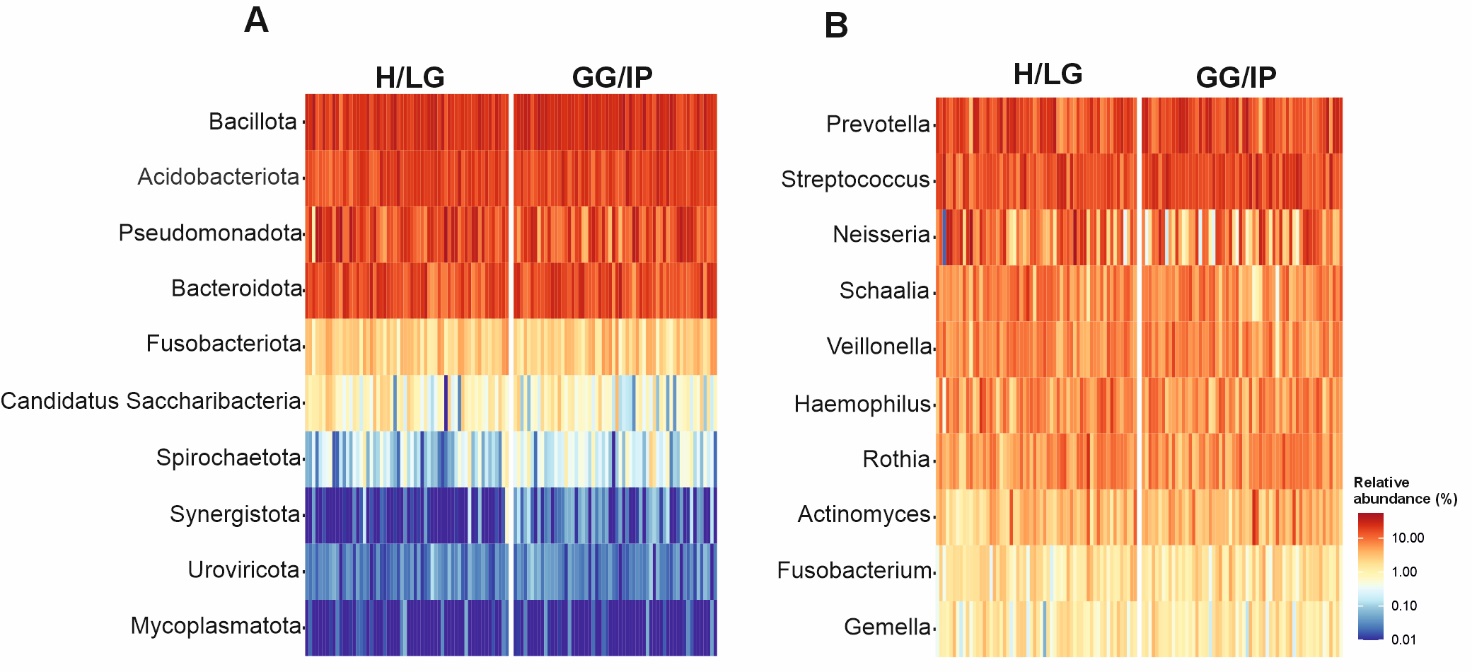


**Fig. S1.** Relative abundance of the top 10 phyla and genera in H/LG and GG/IP groups. H/LG: healthy and localized gingivitis, GG/IP: generalized gingivitis/initial periodontitis.
